# Views upon the Statics of the Human Chest, Animal Heat, and Determination of Blood to the Head

**Published:** 1844-01

**Authors:** 


### Art. VIII.

*Views upon the Statics of the Human Chest, Animal Heat, and Determination of Blood to the Head.* By Julius Jeffreys, f.r.s. &c.—*London*, 1843. 8vo, pp. 234.

Mr. Jeffreys is very generally known to the profession as the ingenious inventor of the "Respirator." He is the author of various scientific memoirs of merit, and, among others, of an interesting series of papers on "artificial climates," which appeared in the Medical Gazette, (1842, Nos. xxii to liii.) The work before us contains an exposition of those views of the physiology of respiration, which, among other practical results, led to the construction of the instrument referred to. It contains also the views of animal heat, and of determinations of blood, which the author has been led to adopt; illustrated by a number of interesting observations and reflections made during a long residence in tropical climates. These refer chiefly to the effects produced by the peculiar habits of different nations in respect to their food and clothing; to the phenomena developed during remarkable states of the atmosphere as regards its temperature, moisture, and density.

Part I is devoted to the statics of the chest. The author divides the air of respiration into four quantities: 1st, the *residual air*, or that which cannot be expelled from the lungs, but remains after a full and forcible expiration; 2d, the *supplementary air*, or that which can be expelled by a forcible expiration, after an ordinary out-breathing; 3d, the *breath*, or tidal air; and 4th, the *complementary air*, or that which can be inhaled after an ordinary inspiration. The volume of these four quantities, Mr. Jeffreys estimates as follows;

| | | |
|---|---|---|
| Residual air | 120 | cubic inches. |
| Supplementary ditto | 130 | " |
| The breath | 26 | " |
| Complementary air | 100 | " |

This estimate gives 250 cubic inches as the average volume which the chest contains after an ordinary expiration, consisting of residual and supplementary air, and constituting, together, what Mr. Jeffreys denominates the *resident air.* The estimate of the first quantity is that of Dr. Bostock. The estimates of the supplementary air and of the breath differ somewhat from those which have been stated by other observers. The supplementary air was estimated by Jurin at 220, and by Dr. Bostock at 160 or 170 cubic inches; by John Bell, on the other hand, it was computed to be as low as 70. From the very careful experiments of Dr. Menzies, the volume of the *breath* was estimated by him to average 40 cubic inches; an estimate which Mr. Jeffreys justly ascribes to the fact that the observations were made under circumstances which induced laborious breathing, and which was therefore too high. The observations of Mr. Jeffreys on this point appear to have been made with much care and judgment, and probably approximate as nearly as can be expected to the truth.

The author takes considerable credit to himself for pointing out that the air of respiration, or the breath, can have no *direct* concern in the oxydation of the blood, that it can only renew in part the resident air, and by gradual admixture with it, during successive respirations, ultimately reach the air-cells. He asserts that physiologists have always spoken of the *air of respiration* as acting directly upon the blood in the air-cells, and of it, "when breathed out, as having given up a portion of its oxygen to the blood, and having received from the blood, carbonic acid and watery vapour."

Without detracting at all from the merit of Mr. Jeffreys for the clear and accurate description which he gives of the mode in which the ventilation of the lungs is carried on, and for the important bearings upon the physiology and pathology of the respiration which he points out in connexion with these facts, we cannot admit that physiologists have been guilty of the error attributed to them. The air of the breath has been experimented upon, and regarded only as affording an estimate of the average changes which must take place in a given time in the lungs, and may have been, for the sake of brevity, talked of as if it were the direct and immediate agent of arterializing the blood; but in the words (e. gr.) of Majendie, "the portion of air expired is not exactly that which was inspired immediately before, but a portion of the mass which the lungs contained after inspiration;" and, "inspiration and expiration are intended to renew in part the considerable mass of air contained by the lungs."

The theory of animal heat adopted and illustrated by Mr. Jeffreys is that of Crawford, as modified by Lavoisier. From a consideration of the diet of the inhabitants of the tropics, he concludes that a much greater quantity of heat would be generated in their bodies, than could possibly be got rid of in the high temperatures and moist atmospheres to which they are exposed. From this he infers that the manner and intensity of the union of carbon and hydrogen with oxygen, to form carbonic acid and water, must be regulated by the vital powers in such a manner as "to yield very different quantities of heat at different times." (p. 78.) This conclusion we fear, if it follows necessarily from the premises, only proves the insufficiency of that theory of animal heat which requires to be so propped up.

Our author's theory of animal heat leads him into some curious speculations on the sources of nutrition. Conceiving that in herbivorous animals in hot climates, and in the rice-eating Hindoo, the non-nitrogenous principles of their food would be insufficient for the renewal of their animal fabrics; and that at the same time, to obtain the little azotized matter which it can afford, they would be compelled to consume an unmerciful quantity (considering the heat of the climate) of fuel in the "heat-producing carbon and hydrogen" of their vegetable food; he comes to the conclusion that the nitrogen of the atmosphere is laid hold of by the vital power, and by its means the non-nitrogenous principles of vegetable food are *assimilated.* This hypothesis, ingeniously as it is advocated, calls for more evidence than has been advanced in support of it in the work before us. But we think, on the other hand, that the doctrine of Liebig cannot be regarded as by any means satisfactorily demonstrated; and that many of the objections urged by Mr. Jeffreys should be weighed better than they have yet been, ere we assent to the principle, that no conversion of a non-azotized to an azotized compound ever takes place in the animal body. That this conversion, however, if it should be demonstrated to take place, is effected by any other than *chemical* agency, we cannot by any means admit. Much as we respect the "vital power," we have our doubts of its ability to do several things attributed to it in the work before us. While we protest, as we have always done, against the too-prevalent error at the present day of regarding the human body as a mere laboratory or machine, we would guard equally against resuscitating, for the sake of an hypothesis, the *vis medicatrix naturæ* of former times, and ascribing to it the discretionary power of regulating entirely the operations of the living body, of uniting or separating elements "*at will,*" depositing or removing substances, forming them out of their elements, and tearing them asunder when formed. (p. 122.) The vital power has no will or discretion in the case; the will and discretion are in the arrangement of the vital laws, which are doubtless as definite and determinate in their operation as those of chemical action.

In a chapter devoted to the examination of the function of the capillaries, Mr. Jeffreys endeavours to explain the changes produced in inflammation, by *vermicular contractions* of those tubes. Just as a leech fills itself by vermicular contractions, so, he conceives, the capillaries of an inflamed part fill themselves with blood; and warm fomentations resolve the inflammation by diminishing the tone and action of the capillaries, so that they no longer force blood in undue quantity into themselves. The theory and illustration appear to us to be alike unsatisfactory. If a leech were a tube, patent during the time of its contractions at either end, we question whether it would become distended; nor can we conceive how the capillaries would become distended by similar movements, unless there was added to the hypothesis a "tonic spasm," or sphincter, on some point of the vessels.

In Part III, "On the dissipation of animal heat, and its influence in producing local determinations of blood, especially to the head," Mr. Jeffreys, in pointing out the secondary effects of cold and warmth, boldly controverts the common axiom "keep the head cool and the feet warm." He points out that the Hindoos, who habitually reverse this rule, are remarkably exempt from cerebral affections, in a climate where Europeans are peculiarly liable to them; and that persons who habitually act on the vulgar axiom produce, by preventing the proper functions of the parts, and the usual extrication of heat from them, a cold and bloodless state of the extremities; while the head, on the other hand, kept bald and cool, displays by its flushings and perspiration an increased tendency to vascular action. The observations are to a great extent true, and deserving of consideration; the axiom we have been accustomed to hear, and to act upon, however, will bear, we think, the test of our author's experience and observation; and is, we believe, the full and proper exposition of the axiom in question, namely, "keep the feet warm *with exercise*, and the head cool *with temperance*."

The work before us, although we have expressed ourselves thus freely on several of the most prominent theories which it propounds, contains much interesting matter for inquiry and reflection. It is the production of one who has enjoyed very favorable opportunities of observing nature in widely different circumstances, and who has brought to bear upon those opportunities the powers of an intelligent and reflective mind, sharpened and chastened by an acquaintance with the gradual progress of physiological research throughout the world. The critique on Liebig's views, contained in the various articles which form the Appendix, is especially deserving consideration; although we think that more extended chemical and physiological information on Mr. Jeffreys's part would have prevented him from making some unfounded attacks upon the doctrines alluded to.

